# Genomic Traces of the Fruit Fly *Anastrepha obliqua* Associated with Its Polyphagous Nature

**DOI:** 10.3390/insects12121116

**Published:** 2021-12-14

**Authors:** Elkin Aguirre-Ramirez, Sandra Velasco-Cuervo, Nelson Toro-Perea

**Affiliations:** Grupo de Estudios Ecogenéticos y Biología Molecular, Departamento de Biología, Universidad del Valle, Cali 760032, Colombia; sandra.velasco@correounivalle.edu.co (S.V.-C.); nelson.toro@correounivalle.edu.co (N.T.-P.)

**Keywords:** adaptation, population differentiation, host plant races, RAD-seq, Diptera, Tephritidae

## Abstract

**Simple Summary:**

Individuals of a polyphagous species, such as *Anastrepha obliqua*, that plague different host plants, present differences at the genome level; these differences are associated with adaptive processes related to the exploitation of the resource and can lead to speciation in sympatry, first with the formation of host races. In this studio, we used pooled RAD-seq to assess genomic differentiation and population structure in sympatric populations of *Anastepha obliqua* that infest three different host plants. The results obtained support the effect of host plants on genomic differentiation in populations of the species *A. obliqua*. We identified a small group of candidate genes that could be under divergent selection, a product of the selective pressure mediated by the host plants.

**Abstract:**

*Anastrepha obliqua* (Macquart) (Diptera: Tephritidae) is an important pest in the neotropical region. It is considered a polyphagous insect, meaning it infests plants of different taxonomic families and readily colonizes new host plants. The change to new hosts can lead to diversification and the formation of host races. Previous studies investigating the effect of host plants on population structure and selection in *Anastrepha obliqua* have focused on the use of data from the mitochondrial DNA sequence and microsatellite markers of nuclear DNA, and there are no analyses at the genomic level. To better understand this issue, we used a pooled restriction site-associated DNA sequencing (pooled RAD-seq) approach to assess genomic differentiation and population structure across sympatric populations of *Anastrepha obliqua* that infest three host plants—*Spondias purpurea* (red mombin), *Mangifera indica* (mango) of the family Anacardiaceae and *Averrhoa carambola* (carambola) of the family Oxalidaceae—in sympatric populations of the species *Anastrepha obliqua* of Inter-Andean Valley of the Cauca River in southwestern Colombia. Our results show genomic differentiation of populations from carambola compared to mango and red mombin populations, but the genetic structure was mainly established by geography rather than by the host plant. On the other hand, we identified 54 SNPs in 23 sequences significantly associated with the use of the host plant. Of these 23 sequences, we identified 17 candidate genes and nine protein families, of which four protein families are involved in the nutrition of these flies. Future studies should investigate the adaptive processes undergone by phytophagous insects in the Neotropics, using fruit flies as a model and state-of-the-art molecular tools.

## 1. Introduction

A fundamental challenge in contemporary evolutionary biology is to understand the genetic mechanisms that determine how organisms can adapt to different environments [1]. Although there is evidence of these adaptive processes in wild conditions [2,3,4], the total understanding of genetic foundations in almost any system remains a challenge. In this sense, phytophagous insects have been characterized as an excellent biological model for understanding the processes involved in adaptation to new ecological environments [5,6,7,8,9,10]. This is mainly due to the close relationship between phytophagous insects and their host plants, which constitute their habitat during all or some stages of their life cycle, their oviposition site, or their food source, a relationship that leads to the selection of adaptations associated with the phenology of the plant [11]. Generally, in this ecological relationship, an optimal use of the available food resource is observed, but many times, the insect can begin to exploit other food resources around it. The opportunity to use new species of host plants can contribute to either the expansion of the distribution range of a species or lead to a process of ecological diversification with the formation of host races [12]. A host race is defined as a population of a species that is partially reproductively isolated from other conspecific populations as a direct consequence of adaptation to a specific host [13]. Conceptually, host races and species reside on different ends of a gene flow continuum, with host races representing the hypothesized incipient stage of ecological speciation and host-associated species its final product [5,14].

An interesting model of phytophagous insects with which to study the effect of host shifts has been fruit flies (Diptera: Tephritidae). This family of flies is currently composed of approximately 4500 species [15], many of which are pests of economic importance worldwide. In addition to their status as pests, tephritids include several species that have been studied by evolutionary biologists to understand the rapid divergence of species in different scenarios, such as ecological speciation in sympatry [5,9,16,17]. Among the genera of the family Tephritidae, *Anastrepha* Schiner is the most diverse and economically important in the Neotropics, with more than 300 species [18]. Few studies assess the effect of host shifts in the genus *Anastrepha* (e.g., [19,20,21,22,23,24,25,26]).

In this study, the study organism was the species *Anastrepha obliqua* (Macquart), the West Indian fruit fly. This species is distributed from northern Mexico to southern Brazil and the Caribbean, and is occasionally found in Texas and California. In addition, it is part of the taxonomic group fraterculus and is considered an important pest of 60 species in 24 plant families of tropical and subtropical areas [26,27]. In the southwest of Colombia, *A. obliqua* has been found to be infesting fruits of mango (*Mangifera indica* L., Anacardiaceae), red mombin (*Spondias purpurea* L., Anacardiaceae), carambola (*Averrhoa carambola* L., Oxalidaceae), and araza (*Eugenia stipitata* McVaugh, Myrtaceae) [24]. Additionally, *A. obliqua* has been reported to infest other fruits, both of commercial and wild interest [28]. This apparently generalist behavior suggests a process of progressive adaptation to new host plants.

Although few studies have been conducted to understand the association of *A. obliqua* with its host plants, some molecular studies have provided relevant information on this topic. For example, Aguirre-Ramirez et al. [24] found differences in the distribution of haplotype frequencies between populations infesting different host plants. Ruiz-Arce et al. [26] analyzed *A. obliqua* populations in the state of Veracruz, Mexico, from five host plant species and 52 geographic collections using data from mitochondrial DNA sequences and microsatellite nuclear DNA markers. They found significant differences between samples collected from different host plants in one locality (Apazapan), which could imply a certain degree of fidelity for the host plant (*S. purpurea* vs. *M. indica* var. Criollo) that restricts the ongoing gene flow between populations.

Traditionally, studies that seek to understand the basis of an adaptive process in nature use common garden experiments, where individuals from contrasting environments are exposed to a common laboratory environment [29] and statistical methods are used to infer the genetic differentiation underlying phenotypic traits [2]. These methods have been increasingly integrated with population-genetic tools to infer adaptation [30]. More recently, advances in genomic technologies have made it possible to identify genomic regions associated with adaptive process, for example, [9,30,31,32,33,34,35,36,37]. Among such genomic approaches are genome scan or outlier detection methods, which allow inferences about adaptive differentiation [38]. In this research, we used the sequencing of DNA fragments associated with restriction sites (RAD-seq) [39], which allows the identification of single-nucleotide polymorphisms (SNPs) throughout the genome that are involved, for example, in the use of food resources [9]. The advantage of this technique lies in its ability to evaluate multiple SNPs from across the genome and identify regions under divergent selection.

Although members of the family Tephritidae have been used as models to understand the effect of host shift on population dynamics and the evolutionary history of populations [33,40], in the genus *Anastrepha* the genetic-molecular mechanisms are mostly unknown. To investigate this issue, we sampled sympatric populations of *A. obliqua* infesting three host plants in the Inter-Andean Valley of the Cauca River in southwestern Colombia and measured the differentiation between populations through a subset of RAD-seq data. Finally, we used three approaches to identify those significantly differentiated loci that are correlated with the use of host plants.

## 2. Materials and Methods

### 2.1. Sample Collection

Fruits were collected from three host plants species infested with *A. obliqua* larvae: mango (*M. indica*) and red mombin (*S. purpurea*) of the family Anacardiaceae, and carambola (*A. carambola*) of the family Oxalidaceae. These three fruits were sampled because:

(i) the red mombin is a native species of America and natural host of *A. obliqua*;

(ii) the mango, a species native to Southeast Asia introduced into Colombia in the 16th to 17th centuries [41], belonging to the same family as red mombin, and a crop of economic importance that in Colombia *A. obliqua* is its main pest;

(iii) the carambola an introduced species around the 18th century and brought to Colombia in 1930 [42], belonging to a taxonomic family different from mango and red mombin, which could have a greater impact on the physiology of individuals, due to the composition being different from the fruits.

These plants were all found in localities of southwestern Colombia located in the north and south of the Inter-Andean Valley of the Cauca River. The reason why this region was chosen is because Aguirre-Ramirez et al. [24] reported that there was no population genetic structure of *A. obliqua* associated with geographic distance in the Inter-Andean Valley of the Cauca River. To choose the populations of fruit flies in the study, the estimated dispersal capacity of wild flies of *A. obliqua* was also taken into account, which has been reported to be approximately 151 m in radius [43].

**Figure 1:**
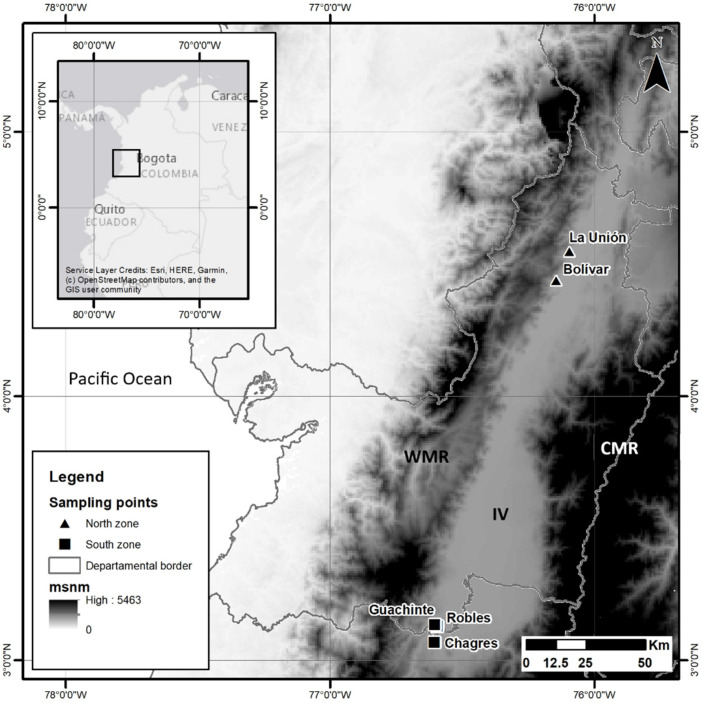
Geographic location of the localities where the fruits infested by the species *Anastrepha obliqua* were collected in southwestern Colombia (the north: localities Bolívar and La Unión and south: localities Chagres, Robles and Guachinte). WMR, Western Mountain Range; IV, Inter-Andean Valley of the Cauca River; CMR, Central Mountain Range.

Based on the above criteria, the north (localities Bolívar and La Unión) and south (localities Chagres, Robles and Guachinte) of the Inter-Andean Valley of the Cauca River were selected as the sampling sites (Figure 1). In each locality, fruits were collected from the three host plants, so each sample consisted of individuals of *A. obliqua* obtained from each host plant for each locality, for a total of six populations (Table 1). Obtaining adult individuals was achieved through the method of Carrejo and González [28]. Fruits were taken that showed signs of infestation, such as soft tissue and/or oviposition holes. They were deposited into a plastic container with vermiculite and covered with very fine porosity (white muslin) cloth and maintained at room temperature until the emergence of adult individuals. Each individual was morphologically identified with the taxonomic key of Caraballo [44]. The identified individuals were preserved in 96% ethanol at −20 °C until DNA extraction.

### 2.2. DNA Extraction, RAD-Seq Libraries Construction and Sequencing

DNA was extracted from the head and legs of adult individuals of *A. obliqua* using the DNeasy Blood and Tissue kit (Qiagen, Valencia, CA, USA). DNA quality was evaluated in a 1% agarose gel. Only nondegraded DNA samples were processed and quantified in a NanoDrop ND-1000 spectrophotometer. Each sample was diluted to 20 ng/µL, re-quantified, and pooled by population (Table 1). Each pooled sample was quantified with a Qubit 4 fluorometer and was equalized to 20 ng/µL for the RAD library construction. RAD libraries and sequencing were carried out by BGI Tech Solutions, Co., Ltd. (Hong Kong, China). Briefly, DNA was fragmented by the restriction enzyme EcoRI. A P1 adapter, including a forward amplification primer, a sequencing primer and a specific barcode, was added to each of the digested DNA pools. The barcoded samples were then pooled and sheared randomly, and a P2 adapter was added to the sheared DNA fragments. DNA with a P1 adapter was selectively enriched by PCR amplification. Finally, DNA fragments of 300–500 bp were gel purified and sequenced on the Illumina HiSeqXTen platform to generate 150 bp paired-end reads.

### 2.3. Data Analysis

To perform the data analysis, the POOLPARTY v.0.8 pipeline was used with some modifications. This pipeline uses best practices in the analysis of Pool-seq data [45,46]. The following is a brief description of each step of the DNA analysis.

#### 2.3.1. Data Processing, Assembly and Alignment

To reduce the error rate towards the 3′ end of Illumina reads, BBMAP v.37.93 (http://jgi.doe.gov/data-and-tools/bb-tools/) (accessed on 14 January 2020) was used. The raw reads were end-trimmed using a minimum Phred quality score of 20 and a minimum read length of 75 bp.

A de novo assembly was performed with RAINBOW v.2.0.4 [47], which is specifically designed to assemble contigs from RAD sequences. This analysis is not implemented in POOLPARTY, so it was necessary to add it to the pipeline. In short, it first clusters reads together that are less than 4 bp apart. These clustered reads are then recursively divided into groups representing individual allele sequences. Individual allele sequences are then assembled and merged into a final set of RAD contigs. Assembly statistics, such as assembly length, longest contig, and N50 and L50 lengths were calculated with QUAST v.5.0.2 [48].

As semi-global alignment and realignment of unmapped reads is recommended for a pooled sample [49], we used the bwa men algorithm of BWA v.07.12 [50] to map the filtered reads onto the reference RAD contigs. During alignment, SAMBLASTER v.0.1.24 was used to mark duplicate read pairs that arise during polymerase chain reaction amplification or sequencing [51]. Duplicate measurements are removed as they can create biased allele frequency estimates in downstream analyses. Finally, the bam files were sorted with PICARD TOOLS v.2.17.11 (http://broadinstitute.github.io/picard/) (accessed on 14 January 2020), and ambiguously mapped and unaligned reads were removed by SAMTOOLS v.1.5 [52].

#### 2.3.2. Genome-Wide Genetic Variation and Population Genetic Structure

To characterize genome-wide patterns of genetic variation and population differentiation, we calculated the nucleotide diversity (Tajima’s *π*) and population mutation rate (Watterson’s Theta, *θ_W_*) with POPOOLATION v.1.2.2 [53]. Because POPOOLATION is not included in POOLPARTY, it was necessary to add it to the pipeline. For this, the recommendations by Kofler [54] were followed, where (i) the bam files of each population were converted to pileup files with SAMTOOLS; (ii) the genomic regions with insertions/deletions (indels) were identified in the pileup files, and a gtf file was generated with the coordinates of these regions using the ‘identify-genomic-indel-regions’ command; and (iii) the gtf file was used to filter the regions surrounding the indels with the ‘filter-pileup-by-gtf’ command. To standardize the sequencing biases, each pileup file was filtered to a uniform coverage (minimum coverage of 20 and maximum coverage of 200) using the ‘subsample-pileup’ command. Finally, *π* and *θ_W_* were calculated across a non-overlapping 1000 bp sliding window with the ‘variance-sliding’ command, taking into account a minimum minor allele count of two. Additionally, the significance for *π* and *θ_W_* differences among populations was first assessed by conducting a one-way ANOVA. As part of a post-hoc analysis, populations were then assigned significance groupings using Tukey’s honestly significant difference tests. One-way ANOVA and Tukey’s test were performed with REAL STATISTICS v.6.8 (http://www.real-statistics.com) (accessed on 14 January 2020).

To determine the degree and pattern of the population genetic structure, the fixation index (*F_ST_*) for each pairwise population comparison was calculated for each SNP as follows: (i) with BCFTOOLS v.1.5 [55], an SNP call was made from the bam files of each population, and a variant call format (vcf) file was created using an SNP quality (SNPQ) of 20, a global minor allele frequency (MAF) of 0.05, and a total depth of coverage (MIDP) of 20; (ii) a mpileup file containing all populations was created with SAMTOOLS, taking into account the positions of the SNPs called with BCFTOOLS. This mpileup file was used to create a synchronized file (sync) with the ‘mpileup2sync’ command of POPOOLATION2 [56]. Finally, the *F_ST_* for each pairwise comparison was calculated at each of the SNPs using the ‘fst-sliding’ command of POPOOLATION2. Confidence intervals for the *F_ST_* estimates were inferred by bootstrapping 1000 times in the *R* package BOOTSTRAP v.2017.2 [57].

To visualize the multilocus patterns of population differentiation, a Principal Coordinates Analysis (PCoA) plot was generated using the R package LABDSV (https://CRAN.R-project.org/package=labdsv) (accessed on 14 January 2020) based on average *F_ST_* values. Additionally, a hierarchical clustering tree was constructed [58] to evaluate the genetic relationships between the six populations analyzed. For this, the covariance matrix of population allele frequencies (Ω) was calculated with BAYPASS v.2.2 under the core model. The Ω was transformed into a correlation matrix using the ‘cov2cor’ function of R [59]. From the constructed correlation matrix, a distance matrix was defined using the ‘hclust’ function of R to perform the hierarchy grouping. Finally, the tree was plotted with the function ‘plot.phylo’ of the R package of APE [60].

#### 2.3.3. Detection of Selection Footprints

Obtaining the SNPs that were likely to be differentiated as a result of selection was done following the current trend in the literature; applying multiple outlier analyses to detect potential outlier loci with increased stringency. The three methods described below were performed on the SNPs identified with POPOOLATION2. First, SNPs falling into the 99.9 percentile of the empirical distribution of each pairwise *F_ST_* were identified as potentially differentiated loci following an empirical outlier detection approach [61].

Second, the identification of overly differentiated SNPs was based on the *XtX* statistics [62], which are analogous to the *F_ST_* values but take into account the relationship between the analyzed populations and the sampling noise in pooled data (variation in the depth of the sequences between populations and SNPs) [62]. The *XtX* values for each SNP were estimated based on the core model implemented in BAYPASS v.2.2 [58] with the default parameters. To calibrate the *XtX*, a pseudo-observed dataset (POD) was simulated using the R function ‘*simulate_baypass*’. SNPs with *XtX* estimates greater than the 99.9% threshold determined from POD were identified, resulting from adaptative divergence [58].

Finally, we relied on the Bayes factor (BF) calculated under the auxiliary covariate model (AUX), also implemented in BAYPASS, to identify differentiated SNPs associated with the host plants. The AUX model involves the introduction of a binary auxiliary variable to classify each locus as associated or not. This allows us to easily compute posterior inclusion probability (and BF) for each locus while explicitly accounting for multiple testing issues. For each SNP, the Bayes factor was expressed in deciban units (*dB*) via the transformation 10log_10_ (BF). As a decision rule, we then followed the popular Jeffrey’s rule that quantifies the strength of evidence using the criterion of *dB* > 20 as strong evidence [58]. To detect outlier SNPs, we compared the *A. obliqua* populations within the sampled sites (north and south), separately, using core model and AUX model.

To identify candidate genes associated with the outlier SNPs found with the AUX model and at least one of the other two methods, each of the contigs associated with each outlier SNP were annotated with BLAST2GO [63]. We give priority to the AUX model since, of the three, it is the one that allows us to analyze whether a specific SNP can be associated with a given covariate (CaN vs. RmN, CaN vs. MN, RmN vs. MN, CaS vs. RmS, CaS vs. MS and, RmS vs. MS). BLAST2GO was run under default parameters using a blastx search against protein database of arthropods and only considering hits with an e-value cut-off of 1 × 10^−5^. Functional annotation and gene ontology (GO) term mapping was also performed in BLAST2GO under default parameters. Visualization of GO annotations was carried out with WEGO [64].

## 3. Results

### 3.1. Data Processing, Assembly and Alignment

A total of 258.3 million paired reads were obtained from NGS sequencing, with an average of 43 million per population. After filtering, 240.8 million paired reads were retained, with an average of 40.1 million per population (Table 2). The de novo assembly produced 43,175 contig sequences, which were used as reference sequences for the following alignment. The de novo assembly was roughly 25.5 Mb in length (this metric was computed for contigs that exceed the threshold specified in 500 bp), the longest contig was 1699 bp, and the N50 and L50 were 577 bp and 19,332 bp, respectively (Table S1). A total of 124.6 million reads were aligned to the reference sequences, and the number of aligned reads ranged from 20.1 to 21.5 million depending on the population (Table 2).

### 3.2. Genome-Wide Genetic Variation and Population Genetic Structure

To estimate the levels of genome-wide genetic variation, POPOOLATION identified between 481,206 and 565,527 SNPs in the populations evaluated (Table 2). The within-population genome-wide average Tajima’s π and genome-wide average Watterson’s θ_W_ ranged from 0.0105 (populations CaN, RmS and MN) to 0.0108 (population CaS) and from 0.0112 (population CaN) to 0.0117 (population CaS), respectively.

**Figure 2:**
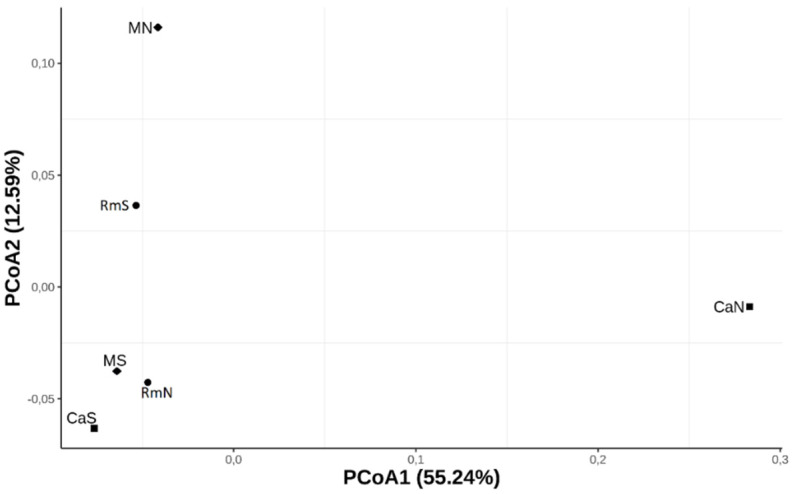
Principal coordinates analysis (PCoA). Plot of the overall average pairwise F_ST_ values of 13,566 SNPs among the six populations of the species *Anastrepha obliqua* of southwestern Colombia.

**Figure 3:**
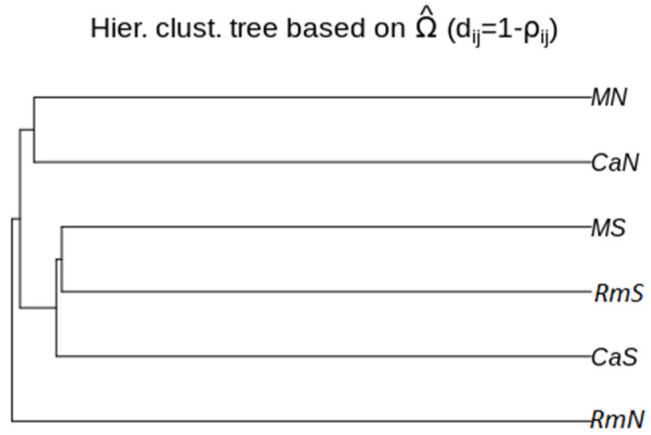
Hierarchical clustering tree showing the genetic relationships among the six populations of the species *Anastrepha obliqua* of southwestern Colombia.

To estimate the levels of genome-wide genetic differentiation between populations, a total of 13,566 SNPs that satisfied our filtering criteria (see methods) were subsampled across all populations with POPOOLATION2. The average pairwise *F_ST_* across all SNPs were similar between the populations. The values varied between 0.0105 (MS vs. CaS) and 0.0162 (CaN vs. CaS). Slightly higher *F_ST_* values were obtained between the CaN population and the other populations (Table 3). This result was consistent with the differentiation pattern observed in the PCoA plot, where the CaN population was the most differentiated from the other populations along the first axis (PCoA1), which explained 55.24% of the genome-wide genetic variation. (Figure 2). Additionally, this axis revealed a geographically ordered pattern where it is observed that the southern populations (CaS, MS and RmS) present a higher degree of genomic relationship with each other than with the northern populations (CaN, MN and RmN). The second axis (PCoA2) explained 12.59% of the variation (Figure 2) and pooled the CaS, RmN, and MS populations, while CaN, RmS, and MN were not pooled with any other population. The reconstruction of the genetic relationships between the populations, based on the hierarchical clustering tree (Figure 3), revealed a differentiation pattern similar to that delineated by the first axis of the PCoA. According to this tree, the MS, RmS, and CaS populations were more closely related to each other and comprised a group separated from the other populations. On the other hand, the CaN and MN populations were grouped, while RmN was separated from them as a basal population for all the others. We can say, then, from the PCoA and the hierarchical grouping tree that the pattern of differentiation of the analyzed populations of the species *A. obliqua* in southwestern Colombia is established mainly by geography.

### 3.3. Detection and Annotation of SNPs under Selection

**Figure 4:**
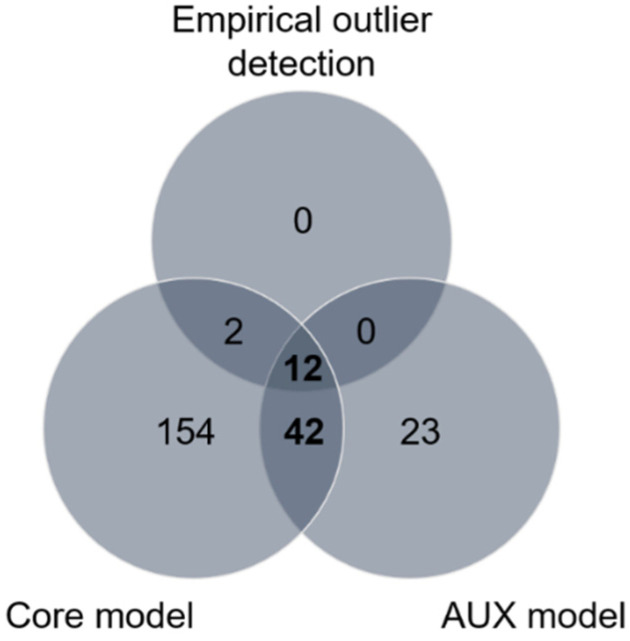
Summary of selection footprint detection using different (empirical, core model and AUX model) methods for the six populations of the species *Anastrepha obliqua* of southwestern Colombia. SNPs identified with AUX model and at least one of the other two methods are considered putative candidate SNPs under host-related divergent selection.

Using the empirical outlier detection approach, 14 SNPs (in 3 contigs) were found at least once in the top 0.1% of pairwise *F_ST_* values (*F_ST_* > 0.0912, Figure S1), which were therefore possible candidates for divergent selection. Additionally, we detected significant selection signatures based on the differences in allele frequencies between populations within the sampling sites (north and south), separately, using two different and robust Bayesian approaches. First, a genomic exploration was performed for differentiated SNPs based on the *XtX* statistic. (the decision criterion to identify SNPs under divergent selection was *XtX* > 6.63, Figure S2). In total, 210 SNPs were detected in 79 contigs. Second, we performed an association analysis to detect SNPs that could be under selection and could be correlated with host plants using the AUX model, also implemented in BAYPASS, with which we identified 77 SNPs (*dB* > 20) in 44 contigs. To our surprise, some overly differentiated SNPs (high values of the *XtX* statistic) were not associated with the use of the host plant (*dB* below expectations), which indicated the presence of other selective pressures. From the results of all three methods, 233 SNPs were identified in 113 contigs. Of these, 54 SNPs in 23 contigs were detected by the AUX model and at least one of the other two methods (Figure 4). The distribution of these 54 SNPs in the population comparisons performed (CaN vs. RmN, CaN vs. MN, and RmN vs. MN for the north; CaS vs. RmS, CaS vs. MS, and RmS vs. MS for the south) showed a higher proportion of SNPs that were associated with host plants in the northern region than in the southern region, suggesting a greater effect of host plant on northern populations of *A. obliqua* (Table S2). The SNPs in the north were different from the SNPs found in the south (Table S2). Four SNPs were recurrent in the north when we compared the MN population with respect to the CaN and RmN populations. In the south, we only found one recurring SNP, between the MS population and the other populations (CaS and RmS) (Table S2).

Of the 23 contigs that contained SNPs that were shown to be correlated with the use of host plants, we identified 17 contigs that presented homologous sequences with the NCBI arthropod protein database in a BLASTx search (Table S3). Of the 17 contigs that managed to be annotated, six could be associated with a GO term (Figure S3). Gene ontology (GO) annotations revealed several loci could be assigned molecular functions of catalytic activity and binding processes and loci that could be related to cellular components, especially membrane components (Figure S3).

**Figure 5:**
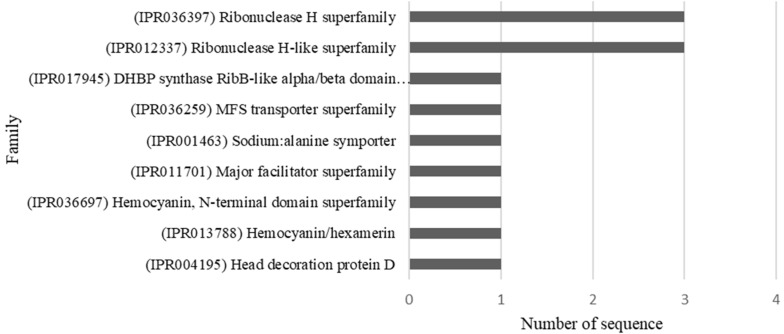
Families of proteins associated with the sequences that contain SNPs that showed signs of being differentially associated to host plants of *Anastrepha obliqua* analyzed.

We also determined the protein families related to the identified sequences. For this, we used the InterProScan tool of the BLASTO2GO program to retrieve information about the protein families (Figure 5).

## 4. Discussion

In this study, we used a pooled RAD-seq approach to evaluate genomic differences between six wild populations of the species *Anastrepha obliqua* associated with the use of host plant. To date, genetic studies that have provided information on the association of *A. obliqua* with its host plants have focussed on the use of data from mitochondrial DNA sequences and microsatellite markers of nuclear DNA [24,26], and there are no analyses at the genomic level in the species *A. obliqua*. This study is the first to provide information at this level on the population differentiation of *A. obliqua* associated with its host plants. The most interesting findings of this work are as follows: (i) the analysis of the genetic differentiation between the populations yielded low values of *F_ST_* based on the genomic SNPs examined. However, (ii) a subset of them showed signs of adaptive differentiation, suggesting the presence of selective pressures in these populations. Of the SNPs identified as being putatively adaptive 54 were associated with host plant specificity. Finally, (iii) some of the adaptive genomic regions linked with host plant specificity are associated with protein families involved in nutrition.

### 4.1. Genome-Wide Genetic Variation and Population Genetic Structure

The *π* and *θ_W_* between the analyzed populations were very similar, with a variation of 0.0105 to 0.0108 for *π* and from 0.0112 to 0.0117 for *θ_W_*. However, we found evidence, with Tukey’s test, of significant differences between the patterns of genomic variation in the populations, for both *π* and for *θ_W_*. According to our analysis, the differences found between the patterns genomic variation were more pronounced between the populations of *A. obliqua* from carambola fruits than between mango and red mombin populations (Table 2). We did not find significant differences between the patterns of genetic variation of *A. obliqua* populations that infest mango and red mombin. On the other hand, the degree of genomic differentiation was generally low (*F_ST_* ranged from 0.0105 to 0.0162) among the populations of *A. obliqua* of different hosts plants species. This result is similar to that found by Bakovic et al. [9] when estimating genomic differentiation in *Rhagoletis cerasi* populations from 2494 SNPs. This fruit fly uses two species of host plants, *Prunus* spp. and *Lonicera* spp., which can be found in sympatry. These researchers revealed that the genomic differentiation between the populations of this species was low (*F_ST_* ranged from 0.004 to 0.143), especially when they compared populations of different host plants found in sympatry. Based on the average pairwise *F_ST_* values obtained, we can suggest that the populations analyzed in this study show low genetic differentiation. The level of genetic differentiation observed is likely due to that ongoing genetic flow persists between the populations or due to a recent descent of the populations.

The population of *A. obliqua* with the greatest degree of genomic differentiation from the others was CaN, since its average pairwise *F_ST_* values were higher. This result is consistent with the differentiation inferred from the patterns of genetic variation of the populations. What could have caused this? Studies of the chemical composition of fruits of different plant species have shown that they can vary widely in both nutrients and secondary metabolites, which could be toxic to fruit flies. For example, Oroño et al. [22] examined the genetic structure of three populations of *A. fraterculus* associated with three species of host plants that were in sympatry and differed in chemical composition, with the aim of testing the hypothesis of sympatric speciation mediated by chemical substances. The researchers found that the adaptive processes related to the plant chemistry of the host plants seem to have promoted genetic differentiation between the populations of *A. fraterculus*, since the individuals analyzed were pooled according to the host plants and, in addition, extremely low gene flow was discovered between populations. In this sense, it is possible that the chemical composition of the fruits of carambola plants, which belong to the family Oxalidaceae, exerts selective pressure on the larvae that feed on the carambola pulp, promoting their genomic differentiation more than the red mombin and mango plants, which are part of the family Anacardiaceae. This differentiable pattern was also found when the bacterial community was analyzed in populations that infest the same three host plants (red mombin, mango, and carambola) in the same geographic region analysed by us [65]. These researchers found that composition of the bacterial community of *A. obliqua* of carambola fruits was significantly different from that of the same fruit fly in mango and red mombin. On the other hand, Aguirre-Ramirez et al. [24] reported the existence of genetic differentiation in *A. obliqua* populations that were associated with different host plants. Particularly for carambola fruits, these authors reported a lower haplotype diversity than was found on mango and red mombin fruits. Because the number of individuals in the CaN population is lower than that of the other populations (see Table 1), we suggest that future research consider the possibility of corroborating the interpretations made here about the CaN population. This will enable researchers to understand if the genetic differentiation of the CaN population due to the low number of individuals sampled, and whether that with a higher sample number the within-population genetic differentiation will be reduced.

In general, we did not observe a genetic structure associated with the host plant. The first axis (PCoA1) of the principal coordinate analysis and the hierarchical clustering tree suggests the possibility of a genetic relationship among the populations of *A. obliqua* that were in sympatry sharing the same geographic region, be it the northern region or the southern region of the Inter-Andean Valley of the Cauca River. This finding could have two possible explanations. First, and according to Hernández et al. [43], the average dispersion distance of wild flies of the species *A. obliqua* is approximately 151 m. We estimate that the distance between the northern and southern localities sampled here is approximately 160 km. Therefore, the mobility of this species, from one region to another, could be limited by its capacity of dispersion, which might explain the closer genetic relationship between the populations of the same region. However, when looking at the characteristics of the Inter-Andean Valley of the Cauca River, we can find a continuum of fruit trees that host *A. obliqua* from north to south, which could favor the gene flow from one region to another. Therefore, the dispersal capacity of *A. obliqua* would not be the only factor that would explain the closer genetic relationship between populations of the same region. The second explanation would be related to the settlement of these populations in different regions, where flies can find their host plants available without having to travel long distances. Our observations differ from the results obtained by Aguirre-Ramirez et al. [24], who did not find a population genetic structure of *A. obliqua* associated with geographic distance between populations from the same geographic sites analyzed in this study. However, it should be noted that our two studies used different molecular techniques. In Aguirre-Ramirez et al. [24], the analyses were performed on the molecular data obtained from two mitochondrial genes (COI and ND6), while in this work, we used the pooled RAD-seq technique to detect divergence patterns throughout the genome.

It should be noted that the main objective of this research was to assess genomic differentiation and population structure in sympatric populations of *A. obliqua* that infest three different host plants. Additionally, we tried an isolation by distance test as described in [66], but it was not possible since our experimental design did not allow it. To perform an isolation by distance test it is necessary to determine the geographical distance and genetic distance between the populations analyzed; with these variables it is possible to estimate the regression equation. However, we have genetic differences between the different populations but we only have a single reference of geographic distance between all populations, which corresponds to the geographic distance that we have estimated from north to south. We consider that the results obtained from the PCoA and clustering tree could guide new research works where the isolation by distance test is considered and compared with results obtained here. According to Doellman et al. [67], in the case of relatively high levels of gene flow, it would be expected that populations associated with host plants would generally be genetically pooled according to geography, with a subset of the genome that would show differentiation related to host plant. Our results agree with the patterns observed by Doellman et al. [67] and with the findings of Ruiz-Arce et al. [26], who worked with populations of the species *A. obliqua* in Veracruz, Mexico. They mention that the genetic structure of populations of the species *A. obliqua* in this locality is determined more by geography than by the host plant.

### 4.2. Detection and Annotation of SNPs under Selection

We do not rule out the participation of host plants in the differentiation of populations, since we identified a subset of SNPs that presented differences associated with the host plant. Research on insects phytophages that manage to form host races has helped to understand how the accumulation of small changes in the genome that show some association with the use of the host plant can act in combination to reduce the effective rate of gene flow and promote the formation of host races and subsequent speciation [68]. For this reason, the subset of outlier SNPs associated with host plants that we identified could be considered evidence of the diversification processes mediated by host plants in the species *A. obliqua*.

We identified 54 SNPs that showed significant differentiation associated with host plant specificity. BLAST search queries and the annotation of the SNP-containing 23 RAD contigs allowed us to identify 17 candidate genes and nine protein families. Among the most interesting annotated protein families, the MFS transporter superfamily and major facilitator superfamily stands out; they are related to the digestive system of herbivorous insects [11] and are involved in the hydrolysis of sucrose and absorption of nutrients [69]. These protein superfamilies could be a key factor that facilitates the adaptation and survival of insects in response to new diets [70]. Additionally, the Hemocyanin, N-terminal domain superfamily and Hemocyanin/hexamerin, which are related to the storage of nutrients, specifically amino acids, for the synthesis of proteins in adults were identified [71]. We also identified the Ribonuclease H superfamily and the Ribonuclease H-like superfamily, which are associated with numerous fundamental processes, including DNA replication and repair, homologous recombination, transposition and RNA interference [72]. Finally, we identified a DHBP synthase RibB-like alpha/beta domain superfamily; this superfamily is related to the enzyme DHBP synthase RibB that catalyzes the conversion of D-ribulose 5-phosphate to formate and 3,4-dihydroxy-2-butanone 4-phosphate [73], and is also related to the YrdC family of hypothetical proteins that are widely distributed in eukaryotes and prokaryotes. YrdC is predicted to be an rRNA maturation factor, as deletions in its gene lead to immature ribosomal 30S subunits and, consequently, fewer translating ribosomes [74].

## 5. Conclusions

In conclusion, we present evidence that supports the effect of host plants on genomic differentiation in populations of the species *A. obliqua*. We cannot say with certainty that this species presents host races. However, our results support the hypothesis that the species *A. obliqua* could be in an incipient stage of population differentiation mediated in part by its host plants. Our results add to a growing list of possible examples of ongoing ecological divergence. We identified a small group of candidate genes that could be under divergent selection; a product of the pressure mediated by the host plants. We believe that in future research, an analysis based on the sequencing of the complete genome of individuals of the species *A. obliqua* would allow us to detect more important genomic regions that support the adaptation of *A. obliqua* to their host plants. Our study is a first step to better understand the population genomics of an important pest of fruit crops, *A. obliqua*.

## Figures and Tables

**Table 1:** Sampling sites and information of the pooled DNA samples of populations of the species *Anastrepha obliqua*.

Host Plant	Locality	Code	Number of Individuals
*Averrhoa carambola*	North	CaN	26
	South	CaS	40
*Mangifera indica*	North	MN	40
	South	MS	40
*Spondias purpurea*	North	RmN	40
	South	RmS	40

**Table 2:** Estimators of genetic diversity in the populations of the species *Anastrepha obliqua* of southwestern Colombia. *π* is the average nucleotide diversity, and *θ_W_* is the average population mutation rate. Letters a, b and c that are found as a superscript in the values *π* and *θ_W_* denote which populations are significantly different as determined by Tukey’s test (*p* < 0.05). Meaning of population codes is provided in Table 1.

Population	Accessions Code	Number of Raw Reads	Number of Aligned Reads	Number of SNPs	*π*	*θ_W_*
CaN	SRR13442704	43,020,774	20,493,546	488,027	0.0105 ^a^	0.0112 ^b^
CaS	SRR13442703	43,183,116	21,589,043	565,527	0.0108 ^b^	0.0117 ^c^
RmN	SRR13442702	43,003,430	20,134,297	481,206	0.0106 ^a^	0.0115 ^a^
RmS	SRR13442701	43,036,924	20,554,814	503,735	0.0105 ^a^	0.0115 ^a^
MN	SRR13442700	43,020,380	20,765,409	524,277	0.0105 ^a^	0.0115 ^a^
MS	SRR13442699	43,048,340	21,039,009	546,956	0.0106 ^a^	0.0115 ^a^

**Table 3:** Average pairwise *F_ST_* values among the six populations of the species *Anastrepha obliqua* of southwestern Colombia based on the 13,566 SNPs identified using POPOOLATION2 (lower diagonal) and 95% confidence intervals (upper diagonal). Meaning of population codes is provided in Table 1.

Population	CaN	CaS	RmN	RmS	MN	MS
CaN	-	(0.0159–0.0166)	(0.0158–0.0164)	(0.0155–0.0161)	(0.0154–0.0160)	(0.0153–0.0159)
CaS	0.0162	-	(0.0106–0.0111)	(0.0105–0.0110)	(0.0109–0.0116	(0.0103–0.0108)
RmN	0.0161	0.0109	-	(0.0117–0.0123)	(0.0112–0.0118)	(0.0108–0.0113)
RmS	0.0158	0.0107	0.0120	-	(0.0109–0.0115)	(0.0107–0.0112)
MN	0.0157	0.0113	0.0115	0.0112	-	(0.0112–0.0118)
MS	0.0156	0.0105	0.0111	0.0110	0.0115	-

## Data Availability

The raw sequence data have been deposited in NCBI’s Sequence Read Archive, and accession code are listed in Table 2.

## Supplementary Materials

The following are available online at https://www.mdpi.com/article/10.3390/insects12121116/s1, Figure S1: Distribution of the average pairwise *F_ST_* values in the 13,566 SNPs obtained with POPOOLATION2, Figure S2: Genomic exploration for loci with a high degree of differentiation according to the *XtX* statistics estimated under the central BAYPASS model. The dashed line represents the significance threshold of 0.1%, Figure S3: GO classification of the six contigs with SNPs correlated with the use of the host plant, Table S1: Assembly statistics, such as number of contigs at certain lengths, total assembly length, N50 and L50 lengths, and number of unknown bases per kilobase (# N’s per 100 kbp) are reported for *A. obliqua*, Tabla S2: BAYPASS analysis using the AUX model to detect SNPs associated with use of host plants: *Mangifera indica* (mango), *Spondias purpurea* (red mombin) and *Averrhoa carambola* (carambola) in six populations of the species *Anastrepha obliqua* located in the north ans south of southwestern, Table S3: BLAST and GO annotation of 23 contigs that contain SNPs that showed signs of being differentially associated to host plants of *A. obliqua* analysed.
